# Tobacco and alcohol use among 11- to 17-year-olds in Germany. Results of the cross-sectional KiGGS Wave 2 study and trends

**DOI:** 10.17886/RKI-GBE-2018-071

**Published:** 2018-06-27

**Authors:** Johannes Zeiher, Cornelia Lange, Anne Starker, Thomas Lampert, Benjamin Kuntz

**Affiliations:** Robert Koch Institute, Department of Epidemiology and Health Monitoring

**Keywords:** TOBACCO USE, ALCOHOL CONSUMPTION, TRENDS, HEALTH MONITORING, KIGGS

## Abstract

Tobacco and alcohol use are among the leading preventable risk factors associated with premature mortality and a variety of diseases that have long-term effects. Although tobacco and alcohol use among adults is widespread in Germany, there is a trend towards lower levels of consumption. The foundations for health-related behaviour in adulthood are set at an early age: young people who use alcohol and tobacco also tend to do so regularly when they reach adulthood. With this in mind, health policies should focus on preventing young people from smoking, and encouraging them to adopt a responsible, low-risk approach to alcohol. This article analyses patterns of tobacco and alcohol use among children and adolescents (aged between 11 and 17 years). It describes the prevalences of tobacco and alcohol use, as well as trends and correlates. The data used in this article was sourced from the second follow-up to the German Health Interview and Examination Survey for Children and Adolescents (KiGGS Wave 2). The survey’s results show that 7.2% of 11- to 17-year-old children and adolescents smoke at least occasionally, with 3.7% doing so daily. The survey also demonstrates that a good half (51.0%) of 11- to 17-year-olds have ever drunk alcohol; at-risk drinking was prevalent among 12.1%, and heavy episodic drinking among 7.0%. The consumption of tobacco and alcohol increases considerably with age. Patterns of at-risk drinking and heavy episodic drinking show gender-associated differences: While more girls than boys practice at-risk drinking, more boys than girls practice heavy episodic drinking. Nevertheless, the KiGGS survey waves demonstrate a highly significant trend towards a decline in tobacco use (KiGGS baseline study 21.4%, KiGGS Wave 1 12.4%). The proportion of 11- to 17-year-olds who have ever drunk alcohol is also declining (KiGGS baseline study 63.9%, KiGGS Wave 1 55.6%). The proportions of at-risk drinking (KiGGS Wave 1 16.5%) and heavy episodic drinking (KiGGS Wave 1 12.0%) decreased as well. The results presented here are in line with findings from other studies that have surveyed adolescent tobacco and alcohol use in Germany, and they underscore the success of preventive measures.

**KiGGS Wave 2**

Second follow-up to the German Health Interview and Examination Survey for Children and Adolescents

**Data owner:** Robert Koch Institute

**Aim:** Providing reliable information on health status, health-related behaviour, living conditions, protective and risk factors, and health care among children, adolescents and young adults living in Germany, with the possibility of trend and longitudinal analyses

**Study design:** Combined cross-sectional and cohort study

**Cross-sectional study in KiGGS Wave 2**

**Age range:** 0-17 years

**Population:** Children and adolescents with permanent residence in Germany

**Sampling:** Samples from official residency registries - randomly selected children and adolescents from the 167 cities and municipalities covered by the KiGGS baseline study

**Sample size:** 15,023 participants

**KiGGS cohort study in KiGGS Wave 2**

**Age range:** 10-31 years

**Sampling:** Re-invitation of everyone who took part in the KiGGS baseline study and who was willing to participate in a follow-up

**Sample size:** 10,853 participants

**KiGGS survey waves**

► KiGGS baseline study (2003-2006), examination and interview survey
► KiGGS Wave 1 (2009-2012), interview survey
► KiGGS Wave 2 (2014-2017), examination and interview survey

More information is available at www.kiggs-studie.de/english

## 1. Introduction

Tobacco and alcohol consumption are among the leading preventable risk factors associated with disease and premature death [1, 2]. It is a well-known fact that even low doses of tobacco smoke are harmful [3, 4]. Furthermore, the nicotine found in tobacco is highly addictive [3]. A number of illnesses, including cardiovascular, respiratory diseases and cancer, are linked to smoking and exposure to passive smoking. In 2013, around 121,000 people died in Germany as a result of smoking; this corresponds to 13.5% of all deaths or one in every seven deaths [5].

Alcohol plays a role in the development of over 200 diseases [6]. Every year, around 14,000 deaths in Germany are entirely attributable to alcohol. Alcohol-related deaths are mainly recorded as ‘alcoholic liver disease’ and ‘mental and behavioural disorders due to use of alcohol’ [7]. Alcohol produces physical and mental reactions that occur during and after consumption [6]. Depending on the concentration of alcohol in the blood, these can lead to short- or long-term physical, mental and societal damage. As such, alcohol affects not only those who consume it, but also other people due to psychological stress, alcohol-related accidents, aggressive behaviour and harm to unborn children. Alcohol can also damage society by placing burdens on the health system and by causing loss of productivity [8]. Finally, a very high level of alcohol in the blood can lead to intoxication, and this can be fatal. A blood-alcohol level of 0.5 mg per millilitre can cause children and adolescents to lose consciousness [6].

Research into adolescent health-related behaviour has identified various individual and societal factors that influence their tobacco and alcohol consumption [9-11]. In addition to age, gender and education (the type of school that a person attends or attended), these specifically include their parents’ and peers’ alcohol- and tobacco-related attitudes and behaviour [12].

Adolescence is a sensitive stage in life, and it is during this time that the foundations are laid for patterns of health-related behaviour in later life. Adolescents often attempt to distance themselves from family or school norms; try out their own behaviour, cross borders and take risks. During this period, their peers become increasingly important (even more important than their parents), and this affects their behaviour, including in terms of their (excessive) use of alcohol or tobacco.

Studies have shown that early use of alcohol or tobacco can encourage regular use in later life [13-15]. The longitudinal analyses of the KiGGS cohort demonstrate that a high proportion of children and adolescents who smoke continue to do so into young adulthood [16]. In a society where alcohol and tobacco use is relatively widespread (despite a trend towards a reduction) [17-19], it is important to encourage young people to develop a responsible, low-risk approach to alcohol. Similarly, young people need to be prevented from starting to smoke wherever possible and those who have already begun to smoke should be encouraged to quit.

Due to the high public health relevance of these issues and the resulting need for action, the cooperation network gesundheitsziele.de has developed the national health targets ‘Reduce tobacco consumption’ [20, 21] and ‘Reduce alcohol consumption’ [22]. Repeated epidemiological studies can be used to assess whether these goals have been achieved. In addition, regularly collected, representative data can provide information about the current situation and trends in tobacco and alcohol use among children and adolescents in Germany. The representative surveys undertaken by the Federal Centre for Health Education (BZgA) [23, 24], the international study Health Behaviour in School-Aged Children (HBSC), which is carried out with the support of the World Health Organization (WHO), [25] and the KiGGS study [26], provide significant sources of data that make this possible.

This article presents current, cross-sectional findings on the prevalence of smoking and alcohol use among children and adolescents from KiGGS Wave 2 and compares developments in tobacco and alcohol use with corresponding indicators from the KiGGS baseline study and KiGGS Wave 1. Finally, it includes an analysis of the relationships between current smoking, school type and smoking by parents and peers.

## 2. Methodology

### 2.1 Study design and study population

The KiGGS study is part of the health monitoring system undertaken at the Robert Koch Institute and includes repeated cross-sectional surveys of children and adolescents aged between 0 and 17 years that are representative for the German population (KiGGS cross-sectional study). The KiGGS baseline study (2003-2006) was carried out as an examination and interview survey; KiGGS Wave 1 was a telephone-based interview survey (2009-2012) and KiGGS Wave 2 (2014-2017) was a combined examination and interview survey. The concept behind KiGGS and its design have been described in detail elsewhere [27-30]. Participants were randomly selected for KiGGS Wave 2 from the population registries held by the 167 representative cities and municipalities that had been chosen for the baseline study. A variety of measures was used to improve participant numbers and sample composition [28, 31]. These include conducting phone calls or home visits to access hard-to-reach groups and to encourage them to participate.

A written questionnaire on child health for the parents and an additional written health questionnaire for children and adolescents aged 11 years or above were used with all of the participants. A total of 15,023 study subjects (7,538 girls, 7,485 boys) participated in KiGGS Wave 2 (response rate 40.1%). The analyses of tobacco and alcohol consumption are based on data from 6,599 participants (3,423 girls, 3,176 boys) aged between 11 and 17 years. Depending on the indicator used, a varying number of participants had to be excluded from the analyses due to missing values.

### 2.2 Indicators

Data was gathered about the smoking-related behaviour and alcohol consumption of 11- to 17-year-old girls and boys for KiGGS Wave 2 using a written questionnaire that was filled out by the respondents themselves. The questionnaire included the question: ‘Do you currently smoke?’ with the following answers: ‘No’, ‘Daily’, ‘Several times a week’, ‘Once a week’ or ‘Less (than once a week)’. In the analyses that follow, respondents who stated that they smoked (at all) are grouped together as ‘current smokers’. However, this group is also subdivided and the prevalences of tobacco use are reported for ‘regular smokers’ (those who smoke at least once a week) and ‘daily smokers’. ‘Regular smokers’ also includes young people who smoke daily. Young people who smoke were also asked: ‘How many cigarettes do you currently smoke?’

Data on the social and environmental factors associated with tobacco consumption by children and adolescents were gathered for KiGGS Wave 2 by asking the parents: ‘Which type of school does your child go to?’ with the following response categories ‘primary school (Grundschule)’, ‘secondary school (Hauptschule)’, ‘middle school (Realschule)’, ‘school with secondary and middle educational program (Schule mit Haupt- und Realschulbildungsgang)’, ‘integrated comprehensive school (Gesamtschule)’, ‘Academic secondary school (Gymnasium) ’, ‘Technical secondary school (Fachoberschule) ’, ‘special school’, ‘other’. In the analyses that follow, secondary schools were grouped dichotomously into ‘academic/technical secondary school’ and ‘Haupt/Real-/Gesamtschule’. In cases where a young person had already left school at the point when the survey was conducted, the highest level of education that she or he had achieved, was used for classification purposes.

In order to gather data on parental smoking-related behaviour, both parents were asked ‘Do you currently smoke?’ (response categories ‘Yes, daily’, ‘Yes, occasionally’, ‘No’). ‘Parental smoking’ was defined as having at least one parent who smoked occasionally or daily.

Irrespective of the parents’ smoking-related behaviour, the parents were also asked whether people smoke in the flat in the presence of their child (response categories ‘Everyday’, ‘Several times a week’, ‘Once a week’, ‘Less often’, ‘Never’). ‘Never’ was categorised as ‘No’; all other responses were categorised as ‘Yes’.

The children and adolescents were asked about the smoking-related behaviour of their close friends: ‘Do friends who are important to you smoke?’ (response categories ‘Yes’ or ‘No’).

As data on smoking status was collected in a similar manner for the KiGGS baseline study, KiGGS Wave 1 and KiGGS Wave 2 [32], these data can be used to asses developments over time and for trend analyses.

Four questions were used to collect data on alcohol consumption. In order to assess the lifetime prevalence of alcohol consumption respondents were asked: ‘Have you ever drunk alcohol?’ (response categories ‘Yes’ or ‘No’). Respondents who answered ‘Yes’ were asked three follow-up questions of the brief alcohol screen AUDIT-C (Alcohol Use Disorders Identification Test-Consumption) in order to collect data on the levels of at-risk alcohol consumption and the distribution of heavy episodic drinking [33]. The first AUDIT-C question asks how often children and adolescents have a drink containing alcohol, such as a glass of wine, beer, mixers, spirits or liqueurs (response categories ‘Never’, ‘Monthly or less’, ‘2 to 4 times a month’, ‘2 to 3 times a week’, or ‘4 or more times a week’. These respondents were then asked the second question: ‘How many drinks containing alcohol do you have on a typical day when you are drinking?’ (response categories ‘1 to 2’, ‘3 to 4’, ‘5 to 6’, ‘7 to 9’, ‘10 or more alcoholic drinks’). Finally, they were asked the third question, which focuses on heavy episodic drinking: ‘How often do you have six or more drinks on one occasion, for example, at a party?’ (response categories ‘Never’, ‘Less than monthly’, ‘Monthly’, ‘Weekly’, ‘Daily or almost daily’). The respondents were also provided with an explanatory note: ‘A drink refers to a small bottle of beer = 0.33 l, a small glass of wine or champagne = 0.125 l, a double spirit or liqueur = 4 cl, or a mixer drink = 1 Alcopop.’ The responses provided to the three questions are scored from 0 to 4, and these scores are then added up. This leads to a final score between 0 and 12 points. At-risk alcohol consumption was assumed to have been identified in cases where girls scored ≥ 4 or boys scored ≥ 5 [33]. The study used the third AUDIT-C question to collect data on heavy episodic drinking. Regular heavy episodic drinking was defined as a report by adolescents that they consume six or more alcoholic drinks on one occasion at least once a month. AUDIT-C was originally designed for screening the adult population, but it is also suitable for use with adolescents [34]. The thresholds for at-risk alcohol consumption are set according to the thresholds used for adults. It is important to remember that adolescents should avoid alcohol as much as possible, and that no threshold values are established for this age group [13, 34].

As data was collected on the lifetime prevalence of alcohol consumption in the same way for all three KiGGS waves, trends can be identified using data from all three periods. However, data on at-risk drinking and heavy episodic drinking was only collected using AUDIT-C for KiGGS Wave 1 [34] and Wave 2. Consequently, trend analyses can only be conducted for these two periods.

### 2.3 Statistical analysis

In the descriptive analysis of tobacco and alcohol use prevalences (frequencies) and mean values with 95% confidence intervals (95% CI) differentiated according to gender, age and survey period are calculated. Differences in socioeconomic status [35] are considered separately in the article Socioeconomic differences in the health behaviour of children and adolescents in Germany. Results of the cross-sectional KiGGS Wave 2 study, which is published in this issue of the Journal of Health Monitoring [36]. The link between current smoking and important factors in the social settings of children and adolescents is initially analysed by comparing smoking prevalences across groups. Logistic regression models were then fitted to estimate age-adjusted odds ratios (ORs) in the first step, which indicate how much higher the odds of smoking are for a particular group compared to a reference group. In the second step a full model containing further variables for adjustment was computed.

The differences that were identified between the groups were tested for statistical significance using Pearson’s chi-squared tests, which were corrected in accordance with Rao and Scott and converted to F-statistics. Regression models (t-tests) were used to test for linear trends between survey waves. A significant difference was assumed to have been identified when confidence intervals did not overlap or if the calculated p-value was less than 0.05.

Cross-sectional analyses were carried out using a weighting factor that adjusted for deviations within the sample from the population structure with regard to age, gender, federal state, nationality and parental education [28]. In addition, a trend analysis was conducted using data from the various KiGGS waves. The analysis used age and gender standardised prevalences identified from data on the distribution found within the German population as of 31 December 2015. The aim was to compensate for possible differences in the age composition of the samples. Analyses were performed using Stata SE 14.2 (Stata Corp., College Station, TX, US, 2015). Stata survey commands were used as part of all of the analyses so as to account for clustering (as participants were selected from specific sample points) and to ensure that weighting was properly implemented during the calculation of confidence intervals and p-values [37].

## 3. Results

### 3.1 Tobacco use

**Figure 1:**
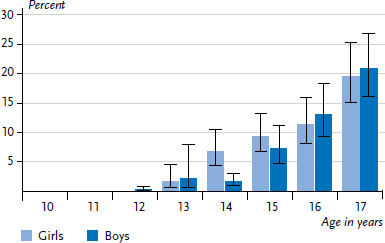
Prevalence of current smoking among 11- to 17-year-olds according to gender and age (n=2,996 girls, n=2,751 boys) Source: KiGGS Wave 2 (2014-2017)

The data from KiGGS Wave 2 show that the vast majority of girls and boys do not smoke. 7.2% of 11- to 17-year-old children and adolescents smoke at least occasionally. 3.7% of adolescents, about half of all smokers, use cigarettes every day; 5.6% do so at least weekly. No significant differences in tobacco consumption were identified between girls and boys (Table 1). The proportion of children and adolescents who smoke increases significantly with age: whereas less than 1% of 11- and 12-year-olds smoke, around 20% of 17-year-olds do so (Figure 1). Adolescents who smoke at least weekly smoke an average of 6.2 cigarettes a day. The average age at which 17-year-olds who regularly smoke took up smoking was 15.3 years. Until the age of 13 years, less than 20% had begun to smoke regularly.

**Figure 2:**
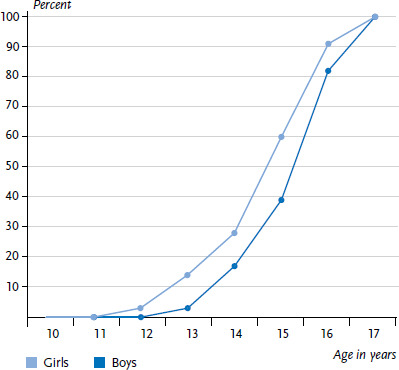
Age at smoking onset among 17-year-old smokers according to gender, cumulated percentages (n=191 girls, n=156 boys) Source: KiGGS Wave 2 (2014-2017)

However, from this age onwards a significant increase in the percentage of smokers can be identified, with girls tending to start smoking earlier than boys (Figure 2).

Previous KiGGS survey waves can be used to study trends in tobacco use. The proportion of young smokers has declined significantly since the KiGGS baseline study (2003-2006). The KiGGS baseline study found that 21.4% of 11- to 17-year-olds smoked at least occasionally. In contrast, the first follow-up survey (2009-2012) found that 12.4% smoked, whereas the current KiGGS wave found that this had dropped to just 7.2%. A similar development can also be observed with regard to daily smoking. The proportion of children and adolescents who smoke daily decreased from 14.2% (KiGGS baseline study) to 5.7% (KiGGS Wave 1) and then to 3.7% (KiGGS Wave 2). Moreover, it is not only the proportion of adolescent smokers that has declined; the average number of cigarettes that adolescents smoke every day has also dropped. Furthermore, the average age of smoking onset among 17-year-olds has risen over time from 14.1 years (KiGGS baseline study) to 15.3 years (KiGGS Wave 2) (Table 2). The data from the current KiGGS wave also show that children and adolescents with a high socioeconomic status smoke less frequently than those with a low or medium socioeconomic status [32, 36].

In addition to findings about the frequency and quantity of tobacco consumption, KiGGS also provides information about the smoking-related behaviour of adolescents’ family and peers (Table 3). 30.6% of girls and boys aged between 11 and 17 years have good friends who smoke. 39.0% have at least one parent who smokes, and 11.7% are confronted with people smoking in their presence while being at home.

However, significant differences occur when the smoking status of children and adolescents is analysed in relation to the smoking-related behaviour of their family and peers as well as the type of school they attend (Table 3). Age-adjusted odds ratios show that girls and boys with parents who smoke are twice as likely to smoke as adolescents with non-smoking parents. If good friends of 11- to 17-year-olds smoke, the odds that these individuals will smoke increase by a factor of 21.0 for girls and 18.4 for boys. A statistically significant association can also be observed regarding the type of school an individual attends. Girls and boys who do not attend a academic/technical secondary school have a higher odds of smoking compared to those who do (girls 2.0 times higher, boys 1.8 times higher). Girls who live in homes where people smoke in their presence have an almost twice as high odds of smoking compared to those who live in homes where this is not the case.

Even after controlling for all other variables, the strong effect associated with friends who smoke still persists: Adolescents who have good friends who smoke have higher odds of smoking than girls and boys who do not (girls 13.5 times higher; boys 15.1 times higher). Furthermore, in boys smoking-related behaviour is significantly linked to that of their parents: after mutual adjustment, boys who have one parent who smokes are also more likely to smoke.

### 3.2 Alcohol consumption

**Figure 3:**
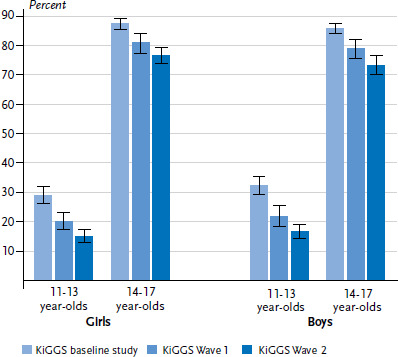
Trends in alcohol consumption (ever) among 11- to 17-year-olds according to gender and age (KiGGS baseline study n=3,274 girls, n=3,426 boys; KiGGS Wave 1 n=2,441 girls, n=2,506 boys; KiGGS Wave 2 n=3,214 girls, n=2,927 boys) Source: KiGGS baseline study (2003-2006), KiGGS Wave 1 (2009-2012), KiGGS Wave 2 (2014-2017)

A total of 51.0% of children and adolescents aged between 11 and 17 years (girls 51.7%, boys 50.2%) have ever consumed alcohol. No significant differences were identified between girls and boys (Table 1). The proportion of girls and boys who have ever drunk alcohol increases with age (data not shown). The figures are 3.7% (95% CI 2.1%-6.3%) for 11-year-old girls and 6.3% (95% CI 4.3%-9.3%) for 11-year-old boys. By the time they reach the age of 17, 87.3% (95% CI 80.5%-92.0%) of girls and 88.5% (95% CI 82.2%-92.7%) of boys have drunk alcohol. The lifetime prevalence of alcohol consumption has steadily declined during the period beginning with the KiGGS baseline study (63.9%) and stretching from KiGGS Wave 1 (55.6%) to KiGGS Wave 2 (51.0%) (Table 2). If the lifetime prevalence of alcohol consumption is calculated for 11- to 13-year-olds and 14- to 17-year-olds and according to gender, it becomes clear that alcohol consumption has particularly declined among 11- to 13-year-olds. This development applies equally to girls and boys (Figure 3). The lifetime prevalence of alcohol consumption is lower among boys from families with low socioeconomic status than among boys from families with a medium or high socioeconomic status. No marked differences were found among girls in terms of the socioeconomic status of the family of origin [36].

**Figure 4:**
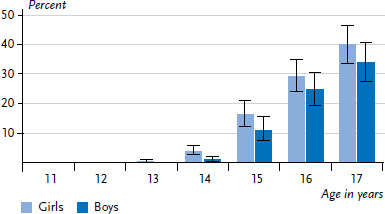
At-risk alcohol consumption (AUDIT-C*) among 11- to 17-year-olds according to gender and age (n=3,133 girls, n=2,836 boys) Source: KiGGS Wave 2 (2014-2017) *Alcohol Use Disorders Identification Test-Consumption

The overall AUDIT-C score shows that 12.1% of 11- to 17-year-olds practice at-risk drinking (Table 1). The prevalence of at-risk drinking increases with age: whereas the prevalence among 11- to 13-year-olds is basically zero, by the time they reach 14 years of age, 3.9% of girls and 1.0% of boys drink at-risk. By the age of 17 years, this rate has risen to 39.9% of girls and 33.8% of boys (Figure 4). Findings about developments over time in terms of at-risk drinking can only be made using data from KiGGS Wave 1 and KiGGS Wave 2. Among 11- to 17-year-old girls and boys, the proportion of at-risk drinkers fell from 17.1% to 13.5% and from 15.8% to 10.8%, respectively, over this period (Table 2).

Heavy episodic drinking (at least monthly consumption of six or more alcoholic drinks on one occasion) is reported by 7.0% of 11- to 17-year-olds. As is the case with at-risk drinking, the prevalence of heavy episodic drinking among 11- to 13-year-olds is basically zero. Among 14- to 17-year-olds, however, 9.2% of girls and 14.2% of boys practice heavy episodic drinking (11.7% of the total). In contrast to at-risk alcohol consumption, an inverse relationship between the genders was identified for heavy episodic drinking: a significantly higher prevalence of heavy episodic drinking was found among male adolescents. The analysis of developments over time shows that the proportion of regular 11- to 17-year-old heavy episodic drinkers fell during the period between KiGGS Wave 1 and KiGGS Wave 2 from 10.2% to 5.6% (among girls) and from 13.8% to 8.4% (among boys).

## 4. Discussion

Data from KiGGS Wave 2 demonstrate that 7.2% of 11- to 17-year-old children and adolescents currently smoke; about half of them daily. With increasing age, the proportion of adolescents that smokes increases significantly. Only slight differences between girls and boys were identified in terms of tobacco consumption. The KiGGS study demonstrates a very clear decline in tobacco use over time: the proportion of current smokers in the most recent survey wave is only one-third of the level identified by the KiGGS baseline study (KiGGS baseline study 21.4%, KiGGS Wave 1 12.4%, KiGGS Wave 2 7.2%). The average number of cigarettes smoked daily by regular smokers has also declined. Moreover, there has also been a further increase in the average age at which 17-year-olds state that they started to smoke.

The results from KiGGS also show a decline in the lifetime prevalence of alcohol consumption among 11-to 17-year-olds from 63.9% (KiGGS baseline study) to 55.6% (KiGGS Wave 1) and 51.0% (KiGGS Wave 2). The levels of heavy episodic drinkers also fell between KiGGS Wave 1 and KiGGS Wave 2 from 12.0% to 7.0%. Despite these encouraging results, alcohol consumption is still highly prevalent among certain age groups. Two in five (39.9%) 17-year-old girls, and one in three (33.8%) boys of the same age consume at-risk levels of alcohol. 16.8% of girls and 30.1% of boys over the age of 17 years still practice heavy episodic drinking at least once a month. A higher proportion of boys consumes excessive amounts of alcohol in the form of heavy episodic drinking, whereas a higher proportion of girls tends to drink at-risk levels of alcohol. It should be noticed that these prevalences are based on the thresholds taken from AUDIT-C, which applies to adults. No limits for low-risk alcohol consumption among adolescents have been established and these groups should avoid alcohol as much as possible.

The results presented here are largely in line with findings from further studies that estimate the prevalence of alcohol and tobacco use among adolescents in Germany [23, 24, 38-41].

The significant decline in the prevalence of adolescent smoking is also evident from the BZgA’s surveys of substance use: over the same period, the proportion of smokers among 12- to 17-year-olds fell from 23.5% (2004) to 7.8% (2015) [23]. The results of the HBSC study also indicate a significant decline in smoking among 11- to 15-year-old pupils since the beginning of the 2000s [38, 39]. A reduction in the prevalence of smoking among adolescents during this period can also be seen in many other similarly economically developed countries in Europe and around the world [40, 42].

Changes in adolescent smoking-related behaviour need to be seen against the background of the intensified tobacco prevention policy that has been implemented in Germany. Since the turn of the millennium, numerous measures have been put in place to curb tobacco use and to protect the population from the health hazards associated with passive smoking. First and foremost, these include significant tax rises between 2002 and 2005 that resulted in substantial price increases, raising the age limit for purchasing and consuming tobacco products to 18 years, widening the advertising ban, broadening warnings on tobacco products and passing non-smoker protection laws at the federal and federal state level. These laws resulted in far-reaching smoking bans in the workplace, public institutions and the catering industry. Moreover, these measures were accompanied by setting- and population-based campaigns and programmes aimed at preventing smoking, especially among young people, and helping smokers to quit [3, 43-44]. International agreements also had helped push forward the advances made in tobacco prevention policy in Germany: the Framework Convention on Tobacco Control (FCTC), which was negotiated under the auspices of the WHO, contains a comprehensive catalogue of tobacco prevention measures that are to be implemented by its member states. The FCTC was ratified by Germany in 2004 [3]. At the same time, some of the measures that Germany has put in place, such as the introduction of ‘shock photos’, are based on binding requirements drawn up by the European Union as part of its regulation of tobacco products (European Tobacco Products Directive) [45].

Even though it is difficult to quantify the preventive effects of individual measures, these measures are likely to have significantly contributed to the fact that a lower proportion of children and adolescents currently smoke in Germany [46, 47].

In addition to prevalences and temporal developments in smoking-related behaviour, the results presented here demonstrate the relationship between adolescent tobacco use and the tobacco-related behaviour of their parents, and, in particular, the tobacco-related behaviour of their peers. Numerous studies have demonstrated the substantial influence that peers and friends have on adolescent smoking-related behaviour [9, 48-50]. Other studies have also shown that adolescents are by comparison more influenced by their peers than their family during this phase of life [49, 51]. Nevertheless, reviews conclude that parental tobacco consumption also has a moderate effect on adolescent smoking-related behaviour [51, 52]. Overall, however, the link between adolescent smoking and the smoking-related behaviour of their friends and family is based on complex processes of socialisation and selection, and claims of a unicausal link lack a sufficient basis [53]. Until now, very few studies are available on the relative impact that best friends, peer groups, and group affiliation have on overall smoking-related behaviour. Therefore, more research is needed into this issue. Nonetheless, the results provide evidence of the influence that parents, friends and peers can have on adolescents and this should be considered when designing appropriate measures.

In principle, the findings on adolescent alcohol consumption are also in line with the results of the representative surveys undertaken by the BZgA. These studies show that the lifetime prevalence of alcohol consumption among 12- to 17-year-olds dropped from 75.3% (2005) to 72.6% (2011) to 63.5% (2016) [24]. However, when comparing these data, the slightly different age groups investigated in these studies should be taken into account. Even though different prevalences were identified, both studies still point towards a significant reduction in alcohol consumption for the entire period.

In terms of the prevalence of heavy episodic drinking, the data from the KiGGS and BZgA surveys show similar developments over time: between KiGGS Wave 1 and KiGGS Wave 2, the proportion of at least monthly heavy episodic drinkers (the consumption of six or more alcoholic beverages on one occasion) among 11- to 17-year-old girls and boys dropped from 12% to 7%; similarly, the Alcohol survey confirmed that the proportion of heavy episodic drinkers (young people who have drunk five or more glasses of alcohol on one occasion in the last 30 days) dropped from 15.2% (2011) to 13.5% (2016) among 12- to 17-year-olds [24].

The decline in alcohol consumption observed from the KiGGS data was also demonstrated by the HBSC study, although the indicators used are not directly comparable to those from KiGGS as different questions were posed [54]. However, overall, data from various population-based studies confirm a decline in alcohol consumption among adolescents in Germany. Nevertheless, this encouraging trend should not be permitted to obscure the fact that a high proportion of adolescents of certain age groups and genders still regularly consume hazardous levels of alcohol.

The WHO’s Regional Office Europe has identified 10 areas for action in its Action Plan to Reduce the Harmful Use of Alcohol between 2012 and 2020, some of which specifically target young people. These include a minimum purchase age of 18 years, reducing the availability of alcohol and the opening hours of places selling alcohol, restrictions on alcohol marketing, and implementing pricing measures, such as tax increases [55].

The availability of alcohol to young people is regulated in Germany by § 9 Jugendschutzgesetz (§ 9 of the Protection of Young Persons Act). The act prohibits the provision of spirits or drinks containing spirits to young people. Other alcoholic beverages (beer, wine, and drinks similar to wine including sparkling wine) can only be provided to young people aged 16 years or over. In addition, § 6 des Gaststättengesetzes (§ 6 of the Restaurant Code) stipulates that restaurants must sell at least one soft drink at the same price as the cheapest alcoholic beverage. This policy aims to ensure that alcoholic drinks are not merely consumed because they are cheaper than soft drinks. However, these regulations require effective control as well as awareness and information on the part of the stakeholders for them to be effective. The campaign ‘Jugendschutz: Wir halten uns daran’ by the Federal Ministry for Family Affairs, Senior Citizens, Women and Youth is important in this respect. In accordance with the WHO recommendations, the German Centre for Addiction Issues states that children and adolescents under the age of 18 years should not drink alcohol and that the protection of minors needs to be extended to the age of 18 [56].

Scientific evidence demonstrates the relationship between alcohol prices and alcohol consumption: higher prices lead to a decline in consumption, and this is most evident among price-sensitive groups such as adolescents [57-59]. Taxes can have a strong influence on alcohol prices [59]. However, a selective increase in individual taxes is unhelpful because it creates a risk that consumers will merely switch to other alcoholic drinks. In Germany, spirits, sparkling wines, beer, alcopops and intermediate products are subject to different excise duties. Wine is not subject to a separate excise tax, and the tax rate on beer is only slightly higher than the minimum rate stipulated by the EU. Overall, Germany has far fewer restrictive measures in place on alcohol consumption than other countries [60].

Behaviour-related preventative measures are aimed at educating young people about the dangers of alcohol and encouraging them to deal responsibly with the issue of alcohol. ‘Alkohol – Kenn dein Limit‘ was the motto of a campaign run by the BZgA aimed at young people about the responsible use of alcohol that specifically targeted young people aged 16 years or older and young adults. The BZgA’s campaign ‘Null Alkohol – volle Power’ aims to encourage children and adolescents aged 16 years or below to adopt a critical approach to alcohol use and to delay their entry into alcohol consumption. The alcohol prevention project ‘HaLT - Hart am Limit’ is particularly aimed at young people who have already began found to be consuming hazardous levels of alcohol, such as through hospitalizations due to alcohol poisoning.

In contrast to the issue of smoking-related behaviour, the KiGGS data faces a limitation when it comes to alcohol use as no information was collected about alcohol consumption by family members and peers. In addition, no data was gathered about the times when alcohol was consumed. Sports associations are considered to be relevant settings for preventative measures in terms of encouraging young people to deal with alcohol responsibly and in a low-risk manner [61]. The first analyses that have been undertaken using data from KiGGS Wave 2 on the links between alcohol consumption and physical activity in sports clubs among 14- to 17-year-old girls and boys show that (once the socioeconomic status of the family and a recent family history of migration have been taken into account) boys who do sports in a sports club are twice as likely to consume at-risk levels of alcohol as boys who do not (data not shown). No corresponding relationship was found among girls. In this regard, the BZgA has established the action alliance ‘Alkoholfrei Sport geniessen’. The alliance has joined the German Olympic Sports Confederation, the German Football Association, the German Sports Youth and other sports federations [62]. The alliance aims to empower children and adolescents in their personal development and to enable them to cope with their lives without turning to addictive substances. Coaches in sports clubs act as essential role models in this respect. Finally, the alliance is aimed at ensuring that the people responsible in sports clubs are made aware of this issue.

Another limitation to this study lies in the survey mode used by KiGGS. Information provided by the respondents on their own alcohol and tobacco use may be biased as it is possible that the respondents provided socially desirable responses. In addition, different survey modes were implemented for the various KiGGS waves: whereas the KiGGS baseline study and Wave 2 collected data using questionnaires that the respondents filled out themselves, KiGGS Wave 1 opted for a telephone survey. As a result, the stronger tendency towards providing socially desirable responses during telephone interviews, which is known from methodological research, may have produced a response bias [34, 63]. Be this as it may, the trends that have been demonstrated are very similar to those from other surveys that have been undertaken of the population in Germany, and no methodological changes were put in place for these studies. In addition, the cross-sectional analyses that were undertaken of the behaviour and associated factors for children and adolescents do not permit causal findings to be drawn. Finally, age, period and cohort effects can only be distinguished to a limited extent when evaluating trends.

## Conclusion

Alcohol and tobacco use among 11- to 17-year-old children and adolescents has dropped significantly over the last decade. The smoking rate identified by KiGGS Wave 2 is already close to the 2020 national health target ‘Reduce tobacco consumption’ (reducing the smoking rate among young people aged 12 to 17 years to below 7% by 2020) [21]. The strategy that has been pursued until now of combining structural and behavioural prevention measures seems to have been effective. For certain age and gender groups, however, there is still a need for prevention and action. Thus, key areas of action set out in the WHO Action Plan to Reduce the Harmful Use of Alcohol have yet to be implemented. Germany lags behind compared to the rest of Europe when it comes to issues such as non-smoker protection, tobacco taxes and widening advertising bans on tobacco products [64]. Since the KiGGS study is designed as a combined longitudinal and cross-sectional survey [65], future surveys will enable participants’ consumption patterns to be tracked over time and analyses to be conducted of the factors that influence alcohol and tobacco use [16, 65].

## Key statements

- 7.2% of 11- to 17-year-old children and adolescents currently smoke; half of them daily (3.7%).
- The proportion of smokers among 11- to 17-year-olds has fallen significantly from 21.4% to 7.2% since the KiGGS baseline study (2003-2006).
- The smoking-related behaviour of children and adolescents is linked to that of their friends.
- A good half (51.0%) of 11- to 17-year-olds have already drunk alcohol, at-risk drinking was prevalent among 12.1% and heavy episodic drinking among 7.0%.
- Since the KiGGS baseline study, the lifetime prevalence of alcohol consumption has fallen from 63.9% to 51.0%. Furthermore, a comparison of data from KiGGS Wave 1 and KiGGS Wave 2 demonstrates a drop in the lifetime prevalence of at-risk drinking from 16.5% to 12.1% and in heavy episodic drinking from 12.0% to 7.0%.

## Figures and Tables

**Table 1:** Smoking and alcohol consumption among 11- to 17-year-olds according to gender and age (n=3,423 girls, n=3,176 boys)* Source: KiGGS Wave 2 (2014-2017)

11-13 Years			14-17 Years		Total	
	%	95% CI	%	95% CI	%	95% CI
**Girls**						
**Smoking, current**	0.6	(0.2-1.6)	11.9	(9.9-14.2)	7.4	(6.2-8.9)
Smoking, regularly	0.2	(0.1-0.5)	8.9	(7.2-10.8)	5.4	(4.4-6.6)
Smoking, daily	0.1	(0.0-0.4)	5.9	(4.6-7.6)	3.6	(2.8-4.7)
Average number of cigarettes smoked^a^					6.3	(5.1-7.2)
**Alcohol consumption, ever**	14.9	(12.7-17.5)	76.7	(73.6-79.4)	51.7	(49.5-54.0)
Alcohol consumption, at-risk drinking	0.1	(0.0-0.5)	22.7	(20.1-25.6)	13.5	(12.0-15.2)
Alcohol consumption, heavy episodic drinking	0.1	(0.0-0.5)	9.2	(7.5-11.3)	5.6	(4.6-6.8)
**Boys**						
**Smoking, current**	0.9	(0.3-2.8)	11.1	(9.4-13.0)	7.0	(5.9-8.2)
Smoking, regularly	0.6	(0.1-3.0)	9.3	(7.7-11.2)	5.8	(4.8-7.0)
Smoking, daily	0.5	(0.1-3.4)	6.1	(4.7-8.0)	3.9	(3.0-5.0)
Average number of cigarettes smoked^a^					6.1	(5.0-7.2)
**Alcohol consumption, ever**	16.5	(14.0-19.3)	73.4	(69.8-76.8)	50.2	(47.7-52.8)
Alcohol consumption, at-risk drinking	0.0		18.3	(15.7-21.3)	10.8	(9.2-12.6)
Alcohol consumption, heavy episodic drinking	0.0		14.2	(12.1-16.5)	8.4	(7.1-9.8)
**Total**						
**Smoking, current**	0.7	(0.3-1.6)	11.5	(10.1-13.1)	7.2	(6.3-8.2)
Smoking, regularly	0.4	(0.1-1.4)	9.1	(7.8-10.5)	5.6	(4.8-6.5)
Smoking, daily	0.3	(0.1-1.5)	6.0	(5.0-7.2)	3.7	(3.1-4.5)
Average number of cigarettes smoked^a^					6.2	(5.4-7.0)
**Alcohol consumption, ever**	15.7	(13.9-17.7)	75.0	(72.6-77.3)	51.0	(49.1-52.8)
Alcohol consumption, at-risk drinking	0.1	(0.0-0.2)	20.5	(18.6-22.6)	12.1	(11.0-13.4)
Alcohol consumption, heavy episodic drinking	0.1	(0.0-0.2)	11.7	(10.4-13.2)	7.0	(6.2-7.9)

CI = Confidence interval

^*^ Case numbers for all participants in the age group 11 to 17 years without missing values for the individual indicators on substance use

^a^ For those who smoke at least weekly; due to the limited number of cases, summarised percentages are shown for the age group 11 to 17 years

**Table 2:** Trends in tobacco and alcohol consumption among 11- to 17-year-olds according to gender (KiGGS baseline study n=3,320 girls, n=3,492 boys; KiGGS Wave 1 n=2,575 girls, n=2,683 boys; KiGGS Wave 2 n=3,423 girls, n=3,176 boys)* Source: KiGGS baseline study (2003-2006), KiGGS Wave 1 (2009-2012), KiGGS Wave 2 (2014-2017)

KiGGS baseline study			KiGGS Wave 1		KiGGS Wave 2		p-value
	%	95% CI	%	95% CI	%	95% CI	
**Girls**							
**Smoking, current**	21.6	(19.9-23.3)	12.2	(10.5-14.2)	7.4	(6.2-8.9)	< 0.001
Smoking, daily	13.8	(12.3-15.4)	5.5	(4.3-7.0)	3.6	(2.8-4.7)	< 0.001
Average number of cigarettes smoked^a^	8.1	(7.4-8.8)	6.4	(5.1-7.7)	6.3	(5.1 – 7.2)	0.001
Average age when 17-year-old smokers began to smoke	14.2	(13.9-14.4)	15.0	(14.6-15.4)	15.0	(14.7-15.4)	< 0.001
**Alcohol consumption, ever**	63.5	(61.6-65.5)	55.9	(53.2-58.5)	51.7	(49.5-54.0)	< 0.001
Alcohol consumption, at-risk drinking			17.1	(15.0-19.4)	13.5	(12.0-15.2)	< 0.01
Alcohol consumption, heavy episodic drinking			10.2	(8.6-12.1)	5.6	(4.6-6.8)	< 0.001
**Boys**							
**Smoking, current**	21.2	(19.5-23.0)	12.6	(10.9-14.5)	7.0	(5.9-8.2)	< 0.001
Smoking, daily	14.5	(13.0-16.2)	5.8	(4.6-7.2)	3.9	(3.0-5.0)	< 0.001
Average number of cigarettes smoked^a^	9.4	(8.8-10.1)	6.9	(5.8-7.9)	6.1	(5.0-7.2)	< 0.001
Average age when 17-year-old smokers began to smoke	14.1	(13.8-14.5)	15.1	(14.7-15.6)	15.6	(15.3-15.9)	< 0.001
**Alcohol consumption, ever**	64.3	(62.2-66.3)	55.3	(52.5-58.1)	50.2	(47.7-52.8)	< 0.001
Alcohol consumption, at-risk drinking			15.8	(13.8-18.1)	10.8	(9.2-12.6)	<0.001
Alcohol consumption, heavy episodic drinking			13.8	(11.9-15.9)	8.4	(7.1-9.8)	<0.001
**Total**							
**Smoking, current**	21.4	(20.1-22.7)	12.4	(11.2-13.8)	7.2	(6.3-8.2)	<0.001
Smoking, daily	14.2	(13.0-15.4)	5.7	(4.9-6.6)	3.7	(3.1-4.5)	<0.001
Average number of cigarettes smoked^a^	8.8	(8.3-9.3)	6.7	(5.8-7.5)	6.2	(5.4-7.0)	<0.001
Average age when 17-year-old smokers began to smoke	14.1	(13.9-14.4)	15.1	(14.8-15.4)	15.3	(15.1-15.6)	<0.001
**Alcohol consumption, ever**	63.9	(62.2-65.6)	55.6	(53.5-57.7)	51.0	(49.1-52.8)	<0.001
Alcohol consumption, at-risk drinking			16.5	(14.8-18.3)	12.1	(11.0-13.4)	<0.001
Alcohol consumption, heavy episodic drinking			12.0	(10.6-13.6)	7.0	(6.2-7.9)	<0.001

CI = confidence interval

^*^ Case numbers for all participants in the age group 11 to 17 years without missing values for the individual indicators on substance use

^a^ For those who smoke at least weekly; due to the limited number of cases, summarised percentages are shown for the age group 11 to 17 years

**Table 3:** Current smoking among 11- to 17-year-olds according to factors in the social setting (n=2,996 girls, n=2,751 boys) Source: KiGGS Wave 2 (2014-2017)

	Prevalence variable		Prevalence of current smoking		Model 1: age-adjusted		Model 2: mutually adjusted	
	Girls	Boys	Girls	Boys	Girls	Boys	Girls	Boys
	% (95% CI)	% (95% CI)	% (95% CI)	% (95% CI)	OR^1^ (95% CI)	OR^1^ (95% CI)	OR^2^ (95% CI)	OR^2^ (95% CI)
**Type of school**								
Academic/technical	52.8	45.3	5.7	5.7	Ref.	Ref.	Ref.	Ref.
secondary school (Gymnasium/Fachoberschule)	(50.1-55.5)	(42.4-48.2)	(4.2-7.6)	(4.2-7.8)				
Haupt-/Real-/	47.2	54.7	8.0	6.7	**2.00**	**1.84**	1.31	1.56
Gesamtschule	(44.5-49.9)	(51.8-57.6)	(6.3-10.1)	(5.1-8.8)	**(1.37-2.91)**	**(1.14-2.99)**	(0.86-1.98)	(0.96-2.55)
Missing values (n=642)^a^								
**Parental smoking**								
Yes	37.4	40.8	**9.7**	**9.6**	**2.05**	**2.21**	1.39	**2.70**
	(34.9-40.0)	(38.0-43.6)	**(7.7-12.2)**	**(7.2-12.7)**	**(1.41-2.99)**	**(1.39-3.52)**	(0.88-2.18)	**(1.50-4.85)**
No	62.6	59.2	**4.9**	**5.3**	Ref.	Ref.	Ref.	Ref.
	(60.0-65.1)	(56.4-62.0)	**(3.6-6.6)**	**(4.1-6.8)**				
Missing values (n=417)^a^								
**Friends who smoke**								
Yes	29.5	31.6	**21.9**	**22.0**	**21.04**	**18.41**	**13.46**	**15.06**
	(27.4-31.8)	(29.5-33.8)	**(18.4-26.0)**	**(18.6-25.9)**	**(10.88-40.68)**	**(6.73-50.38)**	**(6.39-28.34)**	**(4.99-45.41)**
No	70.5	68.4	**0.8**	**0.8**	Ref.	Ref.	Ref.	Ref.
	(68.2-72.6)	(66.2-70.5)	**(0.5-1.4)**	**(0.3-1.8)**				
Missing values (n=55)^a^								
**Smoking at home in the presence of the child**								
Yes	10.6	12.9	**14.6**	10.4	**2.02**	1.67	1.17	0.66
	(8.8-12.8)	(10.8-15.3)	**(10.0-20.8)**	(6.6-16.1)	**(1.28-3.18)**	(0.91-3.08)	(0.59-2.32)	(0.31-1.41)
No	89.4	87.1	**6.4**	6.7	Ref.	Ref.	Ref.	Ref.
	(87.2-91.2)	(84.7-89.2)	**(5.2-7.7)**	(5.5-8.0)				
Missing values (n=144)^a^								

OR = odds ratio, CI = confidence interval, Ref. = reference, Bold = statistically significant (p < 0.05)

^a^ Only cases with valid data on smoking status were included in the mutually adjusted model; all other cases were included in the multivariate model

^1^ Adjusted for age

^2^ Adjusted for age, school type, parental smoking, friends who smoke, smoking in the home in the presence of the child
